# Advances in the Diagnosis of Equine Respiratory Diseases: A Review of Novel Imaging and Functional Techniques

**DOI:** 10.3390/ani12030381

**Published:** 2022-02-04

**Authors:** Natalia Kozłowska, Małgorzata Wierzbicka, Tomasz Jasiński, Małgorzata Domino

**Affiliations:** Department of Large Animal Diseases and Clinic, Institute of Veterinary Medicine, Warsaw University of Life Sciences, 02-787 Warsaw, Poland; natalia_kozlowska@sggw.edu.pl (N.K.); tomasz_jasinski@sggw.edu.pl (T.J.)

**Keywords:** respiratory system, imaging, spirometry, electrical impedance tomography, impulse oscillation system, horse, poor performance

## Abstract

**Simple Summary:**

Respiratory problems are common in horses and are often diagnosed as a cause of poor athletic performance. The basic diagnostic techniques of the equine respiratory tract examination are not always sufficient for a complete diagnosis of the disease, its exacerbation, remission, or response to treatment. Therefore, advances have been introduced in the diagnosis of equine respiratory diseases. Among them, we can distinguish the high-resolution imaging modalities like computed tomography (CT) and magnetic resonance (MR) imaging. These techniques have revolutionized the capability of visualizing detailed anatomy of the upper respiratory tract, offering the practitioners an advanced view of airway pathology and allowing for appropriate management planning. On the other hand, the pulmonary function tests (PFTs), which provide sensitive assessment of small functional changes in the lungs, are able to comprehensively characterize the mechanics of the respiratory system. Spirometry and impulse oscillation system (IOS) analyze intra-breath respiratory mechanics, while electrical impedance tomography (EIT) measures changes in lung conductivity. These methods may be successfully applied to detect airway obstruction and mechanical inhomogeneity in breathing patterns. Presented advanced diagnostic techniques comply with owners’ and trainers’ requirements for accurate and early diagnosis of respiratory tract disorders. This paper reviews advantages, disadvantages, and clinical applications of the advanced diagnostic techniques of the equine respiratory tract.

**Abstract:**

The horse, as a flight animal with a survival strategy involving rapid escape from predators, is a natural-born athlete with enormous functional plasticity of the respiratory system. Any respiratory dysfunction can cause a decline in ventilation and gas exchange. Therefore, respiratory diseases often lead to exercise intolerance and poor performance. This is one of the most frequent problems encountered by equine internists. Routine techniques used to evaluate respiratory tract diseases include clinical examination, endoscopic examination, radiographic and ultrasonographic imaging, cytological evaluation, and bacterial culture of respiratory secretions. New diagnostic challenges and the growing development of equine medicine has led to the implementation of advanced diagnostic techniques successfully used in human medicine. Among them, the use of computed tomography (CT) and magnetic resonance (MR) imaging significantly broadened the possibilities of anatomical imaging, especially in the diagnosis of upper respiratory tract diseases. Moreover, the implementation of spirometry, electrical impedance tomography (EIT), and impulse oscillation system (IOS) sheds new light on functional diagnostics of respiratory tract diseases, especially those affecting the lower part. Therefore, this review aimed to familiarize the clinicians with the advantages and disadvantages of the advanced diagnostic techniques of the equine respiratory tract and introduce their recent clinical applications in equine medicine.

## 1. Introduction

**Figure 1:**
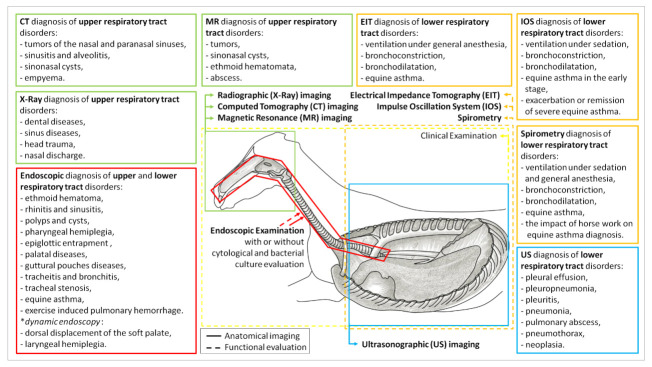
Application of the basic and advanced diagnostic techniques for the anatomical imaging and/or functional evaluation of the areas of the equine respiratory tract. CT—computed tomography; MR—magnetic resonance imaging; EIT—electrical impedance tomography; IOS—impulse oscillation system; X-ray—radiographic imaging; US—ultrasonographic imaging.

Taking a detailed history and performing a good clinical examination is critical to the diagnostic process of equine respiratory diseases. They often provide crucial data which narrows down the diagnostic workup plan and supports the choice of the most appropriate and informative additional examinations. In the case of the equine respiratory tract, observation of the horse from a distance is important to assess the breath rate or any symptoms of respiratory distress. Attention should also be paid to environmental conditions like bedding, ventilation, or access to the pasture. Besides the basic clinical examination, a detailed assessment of the respiratory tract needs to be performed. The respiratory examination is routinely performed in a sequence of inspection, palpation, auscultation, and—when needed—percussion [1,2]. When the basic diagnosis is established, the basic diagnostic techniques of additional examination are routinely used as the initial imaging modality in the evaluation of most common respiratory diseases. Among the basic diagnostic techniques, radiography, ultrasonography, and endoscopy are most utilized in the field of equine practice [1,3,4,5,6,7]. They are often adequate for diagnosis and monitoring of equine respiratory disorders, although advanced imaging techniques are still often required for more detailed assessment of anatomical structures and functional evaluation (Figure 1).

Radiography (X-ray) plays an important role in diagnosing upper respiratory tract diseases affecting the fascial, nasal, and paranasal sinus regions of the horse skull [4]. X-ray is a widely available, portable, inexpensive, and well-tolerated diagnostic technique that can be rapidly and easily obtained in both the field and hospital practices. X-ray of the horse’s head is commonly used for the diagnosis of dental or sinus diseases, head trauma, or nasal discharge [5]. They allow visualization of sinuses, guttural pouches, and the upper trachea, although obtaining diagnostic quality images and interpreting these findings can be an intimidating task [4]. X-ray has inherent limitations when evaluating complex regions such as the skull or thorax due to the complexity and overlap of the anatomic structures. X-ray is of limited value in the evaluation of soft tissue due to reduced tissue resolution. Disease processes are detected radiographically by identifying disrupted or altered contours, size or shape changes, or abnormal radiopacity, thus an adequate coverage of the area of interest is necessary for proper evaluation. These alterations can be difficult to see when there is complex anatomy [8]. To detect changes in radiopacity, mineral loss needs to be advanced at 30% to 50% to detect osteolysis or bone resorption radiographically. There are few pathognomonic findings for many of the disease processes, which can be easily established when carefully assessing the radiographic findings in conjunction with the clinical presentation.

Ultrasonography (US) is another diagnostic tool that allows inexpensive, accessible, real-time, and radiation-free imaging. This technique is implemented in the diagnosis of lower respiratory tract disorders like pleural effusion, pleuropneumonia, pleuritis, pneumonia, pulmonary abscess, pneumothorax, or neoplasia [3]. In general, US provides an assessment of pathological lesions extending mainly to the peripheral lung surface. An accurate characterization of the amount, location, and characteristics of pleural fluid or pleural thickening is easily obtained. Parenchymal lesions can be localized with high accuracy but often appear very similar and cannot be differentiated via an ultrasound examination [9]. During lung inflammation, the most common occurring artifact is comet tail, which can be detected in 91% of horses with equine asthma or exercise-induced pulmonary hemorrhage [10]. However, the comet-tail artifacts are not specific as they usually suggest the presence of a small amount of fluid, without allowing to distinguish its nature (inflammatory fluid, edema, blood, etc.) [10]. The US complements X-ray as it is easier to use, is more sensitive in the detection of smaller amounts of fluid, and provides information about the fluid character, however, it is limited to the lung surface and has a low specificity [11]. For further evaluation of the pulmonary pathology, an ultrasound-guided biopsy can be performed. Additional uses of US include doppler imaging that offers the characterization of the vascularity of masses of the thoracic wall, neck, or pleural space [3].

Rhinoscopy is of great use in the case of an ethmoid hematoma, rhinitis due to foreign body, polyps, and masses causing restricted airflow [12]. It also may be useful in the case of sinusitis when there is a discharge from the nasomaxillary opening. Endoscopy of the pharynx may lead to the diagnosis of pharyngeal hemiplegia, cysts, epiglottic entrapment, or palatal disorders [2]. Visualization of guttural pouches benefits the definition of problems like mycosis, empyema, or tympany [13]. The trachea is examined to ascertain the discharge presence and appearance or to diagnose anatomic defects like tracheal stenosis [14]. Examination of large bronchi provides information about discharge presence and occurring inflammatory process in the lower airway. Under endoscope guidance or separately, broncho-alveolar lavage fluid (BALF) is collected to retrieve fluid and cells lining the distal airways and alveoli. Microscopic evaluation of BALF detects histological abnormalities in horses with pulmonary disease and is commonly used to stage equine asthma (EA) based on a percentage of neutrophils [15]. In clinically healthy young athletic horses, the distribution of nucleated cells in the BALF varies around 60% of macrophages, 34% of lymphocytes, less than 5% of neutrophils, and less than 2% of mast cells or eosinophils [16]. Horses with mild-to-moderate EA usually reveal a mild-to-moderate increase in the percentage of neutrophils, while severe EA is characterized by severe neutrophilia (>20%) [15]. Although the diagnosis of severe cases of EA is relatively easy, it is difficult to diagnose cases in remission or horses with a mild form of the disease [15]. 

Dynamic endoscopy, in some cases, may be the only method for diagnosis of dorsal displacement of the soft palate, especially when the symptoms occur mainly during exercise. It then also provides an assessment of laryngeal hemiplegia during horse movement [7]. Both stationary and dynamic endoscopies are available, inexpensive, and easy to perform under field conditions. 

New diagnostic challenges and the growing development of equine medicine has led to the implementation of advanced diagnostic techniques successfully used in human medicine. Among them, the use of computed tomography (CT) and magnetic resonance (MR) imaging significantly broadened the possibilities of anatomical imaging, especially in the diagnosis of upper respiratory tract diseases. Moreover, the implementation of spirometry, electrical impedance tomography (EIT), and impulse oscillation system (IOS) sheds new light on functional diagnostics of respiratory tract diseases, especially the lower part. Therefore, this review aimed to familiarize the clinicians with the advantages and disadvantages of the advanced diagnostic techniques of the equine respiratory tract and introduce their recent clinical applications in equine medicine.

## 2. Advances in the Anatomical Imaging

### 2.1. Computed Tomography (CT) Imaging of Upper Respiratory Tract

Computed tomography (CT) is an advanced technique established for the detailed imaging of the anatomical structures of the horse’s head and limbs, which nowadays is becoming more available in equine clinics. The CT refers to a computerized X-ray imaging procedure where the patient is radiographed slice by slice, using a rotating, highly collimated, X-ray beam that generates cross-sectional images throughout the area of interest. The CT image is represented by a grayscale map of the tissue’s ability to attenuate X-ray radiation [17]. The CT is useful in imaging primarily bones and structures containing air due to the highest and lowest ability to X-ray radiation attenuation, respectively, however, the soft tissue evaluation by contrasting imaging is also available. The CT provides high quality and resolution of the image and the possibility of three-dimension rotation of each anatomical structure [18]. In the equine respiratory tract, the applicability of CT is limited to imaging of the horse’s head due to the limited diameter of the gantry, in which the examined area must be located during imaging [19]. Smaller horses and foals may fit within the gantry, allowing CT examination of the thoracic cavity and lower airways [20].

#### 2.1.1. Advantages

The CT provides diagnostically important data in situations where X-ray or US has been unrewarding. However, because of costs and the restrictions presented below, it is usually second in line after X-ray or US. The main advantage of CT is the ability to produce high-resolution, three-dimensional, detailed cross-section images [18]. The CT allows more accurate anatomic and morphologic characterization of the anatomic complexity without superimpositions of other anatomical structures within the horse’s body. The CT produces images of the area of interest in various planes. It provides information about the involvement of bones and surrounding structures and may indirectly help the surgery by defining the precise surgical margins of removal [17]. As the images are objective and easy to display and share, using the services of a qualified radiologist from another part of the world is possible [21]. The CT operating systems also provide the possibility to manipulate obtained images to highlight particular structures using grey-level mapping, contrast stretching, histogram modification or contrast enhancement [19]. To evaluate lesions characterized by increased vascular permeability in the equine head, intra-arterial, and intravenous contrast enhancement can be applied [22]. 

#### 2.1.2. Disadvantages

Several problems related to the CT technique that have limited the availability or usefulness in equine practice can be divided into relative and absolute groups. Within the relative group, the high cost of CT equipment and facilities necessary for this imaging is still the main limitation. The monthly maintenance costs of CT scanners are very high and equipment to move anesthetized horses is also expensive [23]. As the cost of CT scanners has come down over the last years, and together with the high demand of horse owners to improve diagnostics, CT imaging is presently not restricted to a limited number of veterinary institutions and large referral centers, and slowly CT examination availability in smaller equine practices has increased [24]. The second relative problem is a need to cooperate with a qualified radiologist. Although performing a CT scan itself is demanding but not very complicated, the viewing and assessment of a large set of highly detailed CT images requires a qualified staff, as for the untrained eye CT imaging may be incomprehensible [24]. Concerning the third relative problem of the imaging artifacts, it should be kept in mind that CT imaging is based on X-ray, ionizing, and radiation with the potential to cause biological effects in living tissues, therefore CT imaging should be done carefully to avoid artifacts and the need for repeated imaging. Within the artifacts which may limit the diagnostic value of CT by masking a lesion or by mimicking a pathologic condition, the partial volume effect and beam hardening should be included [25].

Within the much more important absolute group, the size of the CT scanner is a serious limitation in the use of CT imaging in equine practices. The CT scanners were designed for people, thus in equine practice the diameter of the gantry of the CT scanner, in which the examined area must be located during imaging, ranges from 50–85 cm and allows for insertion of the head, distal limbs, and upper neck of adult full-sized horses [19]. The entire body scan is available only in ponies or foals. Moreover, most available CT scanners require general anesthesia. The horse needs to lie on a table that slides into a gantry of CT scanners. Therefore, the necessity for general anesthesia of the horse during examination is another drawback of CT techniques. When general anesthesia is contraindicated, CT examination will also be contraindicated [18]. The option is the utilization of the modified multidetector systems to perform standing, sedated CT of the equine head. However, the CT examination in the standing position to avoid general anesthesia offers further value, but this technique is still limited to several institutions [18]. 

#### 2.1.3. Clinical Applications

A wide range of diseases affecting the upper respiratory tract have been diagnosed successfully using CT imaging. The intravascular contrast enhancement can be used to differentiate normal soft tissue from lesions based on an alteration in vascular permeability and perfusion which significantly improves the diagnostic value of equine head CT imaging [22]. The CT examination has been proven to be valuable for surgical and treatment therapy planning, especially in progressing diseases where accurate diagnosis and surgical margins play an important role in future prognosis [26]. Precise diagnosis via CT has been emphasized in order to avoid unviable treatment approaches [27]. Malignant and benign tumors of the nasal and paranasal sinuses including squamous cell carcinoma, undifferentiated carcinoma, hemangiosarcoma, nasal adenocarcinoma, myxoma, chondroblastic osteosarcoma, anaplastic sarcoma, and fibro-osseous lesions have been described [28,29,30,31]. Henninger et al. provided detailed CT descriptions regarding the most common features of sinusitis and alveolitis [32]. Diagnosis and surgical treatment of sinonasal cysts have been described [33,34,35]. Solano et al. depicted CT images of empyema, even though endoscopy is the preferred method for the diagnosis process of guttural pouches [24]. Computed tomography has been reported as a tool that overcomes endoscopy and radiography in the evaluation of temporohyoid osteoarthropathy [36,37,38]. Both narrow [39] and larger clinical studies [32,40,41,42] using CT on the equine head have been performed, showing the advantages of this technique in diagnosis and therapy procedures, among which the selected clinical applications of equine CT imaging are summarized in Table 1.

### 2.2. Magnetic Resonance (MR) Imaging of Upper Respiratory Tract

In horses, magnetic resonance (MR), similarly to CT, may support the diagnostic process by providing cross-sectional images of extremities and head. The MR imaging is based on the magnetic dipole nature of the abundant hydrogen protons within tissues and uses the magnetic field and radio waves to create the image of hydrated tissues [19]. The MR images excel in the evaluation of soft tissues, providing anatomic and physiologic information that exceeds tomographic images. The standard image can be acquired in desired orientation, however the commonly obtained planes are sagittal, transverse, and dorsal [44]. Routine MR imaging examinations of the head include three types of pulse-echo sequences: the T1-weighted (T1W) sequence, which is performed before and after contrast administration; proton density (PD); and T2-weighted (T2W) imaging protocols. However, additional imaging sequences may be used [44]. The T1W sequences are based on the longitudinal relaxation properties of tissue and are useful for anatomic detail. T1W images acquired immediately after intravenous contrast administration are compared with identical slices obtained before contrast to detect neovascularisation or dilatation of vessels. Contrast enhancement results in a hyperintense signal in tissues where the contrast has extravasated. PD images are based on the relative concentration of hydrogen protons in different tissues and have the best anatomic detail. T2W images are based on the relaxation interactions between protons [19]. In the equine respiratory tract, the applicability of the high-field closed MR is much more limited than in CT due to the both small gantry diameter and the need to place the center of the imaged object in the center of the gantry [19]. Therefore, the high-field MR imaging of the horse’s head, but no further, is available under general anesthesia only for ponies, foals, or the rostral part of larger horses. The equine low-field MR scanners provide an examination in standing sedated horses; however, the scanner should include a specific system, which is not the same as the small system designed for the equine limb scanning [45,46].

#### 2.2.1. Advantages

The MR provides completely different diagnostically important data than CT, X-ray, or US. The main advantage of MR is the type of acquired data, providing not only excellent anatomic features of soft tissue structures but also functional alterations elusive in other imaging techniques. The MR does not use ionizing radiation, thus no negative biological effects in living tissues have so far been demonstrated [44]. In the equine respiratory tract, MR imaging is particularly useful for identifying space-occupying lesions (tumors, sinonasal cysts, or ethmoid hematomata), where the high soft-tissue contrast of the images is needed to allow differentiation of tissue types and establish an accurate relationship with surrounding structures [42]. The second advantage of MR is the ability to produce high-resolution, three-dimensional, detailed cross-section images which can be accomplished in any plane, without a loss of resolution or quality regardless of the orientation of the horse’s head in the magnetic field [19]. 

#### 2.2.2. Disadvantages

The main disadvantage of the MR technique in equine practice is similar to CT imaging limitations, and also could be divided into relative and absolute groups. Within the relative group, as it was noted for CT that the high cost is still the main limitation, it has to be realized that the costs of MR equipment and facilities are extremely high. The MR magnet, especially in the high-field MR, requires a constant high voltage power supply and continuous cooling with the use of liquid helium, even when no imaging is being performed.

Therefore, the monthly maintenance costs of MR scanners are much higher than those of CT [8]. Moreover, in the case of working in a strong magnetic field, specialized non-metallic equipment for anesthesia, monitoring, and the procedure are needed with special precautions [8], therefore MR imaging is still restricted to a limited number of veterinary institutions and large referral centers. The second relative problem is a need to cooperate not only with a highly qualified radiologist, but also close cooperation with a biophysicist or specialized technical support from the MR service company [47]. In the case of MR, both performing an MR scan and viewing and assessing MR images requires narrow, high-quality MR specialist competencies. For the novice, interpretation of MR images can be more challenging than interpretation of CT scans because of the influence of the many patients- and machine-related factors on the gray-scale display. The principles of image contrast are unfamiliar and follow rules that are not as evident or straightforward [47]. Although three routine sequences are mainly used for MR imaging of horses’ heads, standardization of protocols and specialist technique knowledge is required to achieve high-quality images [19].

As it was mentioned above, the size of the MR scanner is a serious limitation in the use of high-field MR imaging in equine practice, which should be mentioned as an important absolute disadvantage. The gantry diameter allows for the distal limb and rostral part of the head to be introduced in the center for image acquisition [8]. Moreover, the center of the imaged object has to be positioned in the center of the gantry [8], therefore the high-field MR is available only for ponies or foals. The other option is the utilization of low-field, semi-open MR scanners to perform standing, sedated MR of the equine head. However, the quality, resolution, and number of available imaging sequences are much poorer than in high-field MR imaging [47]. Concerning the second absolute problem, the target tissue, it should be kept in mind that MR imaging displays limited clinical use in the lungs and bones evaluation. MR imaging of the foal thorax may be limited due to the sparse soft tissue structures and low proton density for signal production [48]. Moreover, multiple interfaces between air and soft tissue generate susceptibility artifacts and fast signal decay, as in high-field images the field inhomogeneity susceptibility increases with the increase of air it contains. Therefore, the low proton density-dependent artifacts are prevalent, especially on the boundary of air-containing sinuses as well as bone–soft tissue interfaces. The motion of respiratory, cardiac, and vascular systems may also cause artifacts except when the respiratory/ECG gating is available [8]. Finally, in the case of selected horse head disorders, small lesions affecting flat bones of the skull occur which are not detectable on MR images. Thus, for bone imaging CT images are more detailed [19]. However, the decision between CT and MR depends not only on the target tissue, but also, and perhaps most of all, on the time that can be spent on imaging. The MR imaging takes much more time than performing a CT scan, which is not without significance in the case of unstable horses during general anesthesia.

#### 2.2.3. Clinical Applications

Due to the above limitations, a variety of clinical MR applications in equine practice have been reported regarding limbs [49,50,51], while head MR imaging has a limited number of cases. Ferrell et al. presented the clinical application of diagnosis of neurologic diseases in twelve horses, wherein eight of them were successfully diagnosed using MR imaging [52]. Spoormakers et al. assessed the application of MR in equine brain abscesses imaging [53]. A larger study was performed by Manso-Diaz et al., where in 84 clinical cases MR imaging showed the exact location, size of the lesions, and relation to surrounding structures due to neurological, sinonasal, and soft tissue disorders. The disorders of the respiratory tract diagnosed there by MR imaging included sinusitis, dental issues, nasal tumors, nasal septum deviation, and ethmoid hematoma [42]. In the report by Tessier et al., the MR imaging technique was used to diagnose sinusitis, paranasal sinus cysts, ethmoid hematoma, and neoplasia [54]. Garrett et al. reported MR as an advanced imaging method of larynx and pharynx, presenting MR features of laryngeal dysplasia and laryngeal cyst-like malformation [43,55,56]. Potentially, the MR might be a superior imaging technique in the diagnosis of foal lung diseases, following the experiences from human medicine. In human medicine, there are reports where magnetic resonance has been compared with high resolution computed tomography (HRCT) in pneumonia diagnosing. In three reports, the MR imaging had 91% [57], 94% [58], and 95% [59] accuracy, respectively, in the diagnosis of pneumonia compared to HRCT with 100% accuracy. The selected current clinical applications of equine MR imaging of the respiratory tract are summarized in Table 2.

## 3. Advances in the Functional Evaluation

The above basic and advanced imaging techniques predominantly provide data of normality or alterations of the anatomical structures of the respiratory tract. The adaptation of the pulmonary function tests (PFTs) to equine medicine significantly expands the possibilities of the functional evaluation. In contrast to static imaging techniques that allow the direct visualization of the area of interest to diagnose changes like mucus, fluid accumulation, neoplastic changes, or other anatomical abnormalities, dynamic PFTs provide information about the respiratory system in motion. Examples of dynamic measurement are pulmonary resistance and dynamic compliance, which are reliable indicators for airflow obstruction changes [60]. Respiratory abnormalities generally increase respiratory impedance in breathing, and a reduced level of ventilation can be detected objectively by deterioration in breathing mechanics [61]. The PFTs are valuable, noninvasive tools in the investigation and monitoring of breathing mechanics of patients with respiratory diseases. These techniques aid diagnosis, help monitor response to treatment, and can guide decisions regarding further treatment and intervention [62]. However, the PFTs alone cannot be expected to lead to a clinical diagnosis. Further studies on the normal values and appearance of flow-volume curves in equine medicine are required to improve the interpretation of the PETs in horses [63]. Therefore, the PET results should be evaluated in the light of history, physical examination, and diagnostic imaging results.

Among PFS available in human medicine [62], spirometry, electrical impedance tomography, and impulse oscillation systems have been applied to the horses and have enabled tremendous advances in the clinical performance evaluation of the equine athlete [64,65,66]. The selected clinical applications are summarized in Table 3.

### 3.1. Spirometry Evaluation of Lower Respiratory Tract

Spirometry is designed to identify and quantify functional abnormalities of the respiratory tract. In humans, the Global Initiative for Chronic Obstructive Lung Disease recommends spirometry as a diagnostic technique in earlier diagnosis and treatment monitoring in chronic obstructive diseases [78]. In medicine, spirometry provides absolute measures of respiratory function in a simple, reliable, and economical manner. Operating principles are based on three bidirectional pilot flow sensors connected to the face mask that measure breath-by-breath airflow with high resolution [79]. 

Spirometry begins with a full inspiration, followed by a forced expiration that rapidly empties the lungs. Expiration is continued for as long as possible or until a plateau in exhaled volume is reached. Both efforts during inspiration and expiration are recorded and graphed, demonstrating respiratory frequency, tidal volume, peak inspiratory and expiratory flows, time to peak flow, and forced vital capacity, which is an important spirometric maneuver. The forced vital capacity measurement requires the maximal inspiration followed by the rapid expiration, which should be as complete as possible [79]. Such maneuvers have been performed in horses; however, the use of general anesthesia was necessary to avoid interference of conscious respiratory movements with emptying of the lungs [60,80].

In human medicine, spirometry is the gold standard in the diagnosis of lower obstructive respiratory diseases [78], therefore the adaptation of measurement to the horse practice seems to be a promising advancement in the functional diagnosis of equine respiratory diseases. However, in equine medicine spirometry is still restricted to the university centers, and there is a lack of standardized protocol for horse lung investigation [60,64,67,68,69,70,81,82,83,84,85,86].

#### 3.1.1. Advantages

Spirometry provides repeatable and reproducible data of the respiratory function in horses without the need of very expensive and advanced equipment, contrary to CT and MR imaging. Spirometry is a non-invasive technique that does not require the use of ionizing radiation; moreover, it is well tolerated by horses [82,83]. Therefore, spirometry may be conducted on non-sedated horses in standing position [83], however, the application for a horse’s respiratory function monitoring under sedation [70] and general anesthesia [68] is also available. Cooperation with a qualified specialist is required at the beginning for gaining experience with spirometry examination, as the obtained data are simple in interpretation by the practitioners both at rest and during exercise [82,83].

#### 3.1.2. Disadvantages

The main disadvantage of the introduction of spirometry to the equine practice is the necessity of the horse’s cooperation to perform voluntary breathing maneuvers [81]. Because getting a non-sedated horse to maximal inspiration followed by the rapid, deep expiration [79] is almost impossible, in equine medicine spirometry cannot be used in the same manner as in human medicine [78]. Therefore, in horses the spirometry-based pulmonary function tests are dependent on involuntary breathing which can be done with much less cooperation from the horse [67,68,69,70,82,83,84,85,86]. However, the horse still needs to be cooperative with wearing a mask which might be challenging or time-consuming depending on the horse’s activity and temperament [69]. The need to accurately fit the mask to the horse’s head also requires possession of numerous masks so that they can be used for foals, ponies, and full-size horses [86]. Moreover, a certain period of training and acclimatizing horses to spirometric procedures is required to achieve informative data [64]. It should be kept in mind that the horses exhibit a significant respiratory reserve, ventilation, and are subject to rapid change in response to excitement, fear, and other emotional states. One should mention the individual variations and age, sex, and usability-related differences in inflow parameters as the spirometry limitations [64,69,82,83]. Therefore, larger studies to evaluate protocols for equine spirometry are required to move spirometry to the next stage of clinical development in equine medicine.

#### 3.1.3. Clinical Applications

Spirometry has been primarily used to characterize the normal equine tidal breathing flow-volume loop in healthy horses and ponies [82,83]. Afterward, Connally et al. measured the maximal expiratory flow-volume loops in horses exercised on a treadmill, finding no marked difference between clinically normal horses and those with airway obstruction [84]. Further reports revealed changes in breathing strategy and disappearance of biphasic airflow pattern in horses with asthma [69,85]. Herholtz et al. provided strong evidence of the impact of horse work on the differentiation ability in diagnosing different degrees of asthma, as the disease-related differences in spirometry-based measures may consequently be obscured by the type of work undertaken by a horse [69]. Moreover, Burnheim et al. indicated high variability of results across days rather than within traces obtained on a single day with preserved high repeatability and reproducibility [64]. These reports suggest the need for further bigger studies to evaluate protocols for equine spirometry.

Raidal et al. used spirometry for evaluation of the effect of xylazine, acepromazine, and salbutamol on lung function in horses where respiration was significantly reduced by the sedative agents in comparison to salbutamol, where no significant changes were noticed except increased peak inspiratory flow [70], whereas previous studies have suggested that bronchodilation therapy has little effect on healthy horses [86]. Moens et al. investigated the continuous measurement of tidal and minute volume on a breath-to-breath basis in anesthetized horses and concluded that spirometry was useful in the detection of changes like depth of sedation or non-fitted tracheal cuff [68]. 

Among the direct clinical applications of spirometry in the diagnosis of equine respiratory diseases, the spirometry-based early diagnosis of equine asthma was investigated [60,67]. Evans et al. correlated the percentage of neutrophil from tracheal aspirates with spirometry results obtained after exercise and reported that horses with a higher percentage of neutrophils in tracheal aspirates consistently had lower flowtime curves during the second half of both inspiration and expiration [68]. These lower values may be attributed to narrowed airways due to inflammatory exudate, dynamic collapse, and/or airway hyperreactivity associated with asthma. Therefore, the need for pulmonary testing in combination with cytology for compressive diagnosis of equine asthma was strongly suggested [67].

### 3.2. Electrical Impedance Tomography (EIT) Evaluation of Lower Respiratory Tract

Electrical impedance tomography (EIT) is a non-invasive, radiation-free, real-time imaging modality which allows the assessment of lung ventilation and perfusion [87]. The EIT reconstructs a cross-sectional image of the lung’s regional conductivity using electrodes placed circumferentially around the thorax. During EIT examination, a weak alternating current of high frequency and low amplitude is applied between an adjacent pair of electrodes and resulting surface potentials are measured by the remaining electrodes. The measured potentials depend on the tissue bioimpedance and are used to create the functional image of the lower respiratory tract [88]. The tissue bioimpedance changes depending on the fluid content, ion concentration, fat accumulation, or amount of air. Therefore, pathological changes of the tissue composition such as pleural effusion, lung fibrosis, or alveolar fluid accumulation can be easily detected by EIT [87]. 

In human medicine, EIT is frequently used for functional chest examinations, especially for monitoring regional lung ventilation in mechanically ventilated patients, and for regional PFT in people with chronic lung diseases [87]. The EIT is well situated for adults, neonates, and pediatric patients [89]. In the veterinary field, EIT is at an early stage of clinical development, however, in equine medicine the global and regional peak respiratory flows have recently been investigated [73,90,91,92,93]. 

#### 3.2.1. Advantages

The EIT provides functionally unique clinical data of the lower respiratory tract ventilation which is difficult to obtain by other diagnostic techniques. The data are real-time measured and thus reflect continuous ventilation [94]. The EIT allows for the analysis of the individual region of interest in the lung and comparison, for example, of the dorsal with ventral parts of lung regions, the right and left lung, or the same regions that underwent different conditions [65,93]. The electrical currents used by EIT are imperceptible and safe for body surface application with no ionizing radiation exuded [83]. In contrast to CT and MR imaging and similar to spirometry, the equipment and facilities are affordable; easy to implement; well-tolerated by standing, non-sedated horses; and portable, which makes the EIT system suitable for the field conditions [90]. Electrodes are mainly integrated into one electrode belt which makes the application more user-friendly. The EIT working principle does not limit its use to any size of animal, thus the EIT belt can be adjusted to small and full-sized horses [65,95]. Cooperation with a qualified specialist is required during the first period of operation with the EIT belt, but quick and friendly training can also be done online [87]. As reconstruction algorithms have already been adapted to the horse anatomy [65,90,96], the possibility of the EIT application in equine clinical practice has significantly increased. 

#### 3.2.2. Disadvantages

Despite many advantages, the EIT imaging modality shows several limitations. EIT is characterized by very low spatial resolution compared with other imaging techniques, such as CT or MR imaging [93]. The EIT images represent a single cross-section of the thorax [89], whereas CT and MR provide three-dimensional images constructed based on the numerous detailed cross-sections [19]. Other disadvantages are the complexity of EIT data and susceptibility to artifacts. For the understanding of obtained data, specialized training is needed, however, compared to other advanced techniques described here, getting started with the EIT is relatively easy [87]. Concerning the alterations in obtaining data, any patient movement or touching of electrodes during EIT data acquisition leads to the production of the artifacts and thus anatomical and functional distortion. The horse needs to stand quietly, which sometimes may be challenging, and artifacts may still be produced due to gross movement of muscles from muscle fasciculations, interactions with the investigator, sniffing, scratching, or fat tissue accumulation. However, contrary to CT and MR imaging and similar to spirometry, neither general anesthesia or sedation are required [65]. Finally, it should be kept in mind that the EIT in equine medicine is still at an early stage of clinical development, therefore some references and protocols still need to be established [73]. 

#### 3.2.3. Clinical Applications

The first studies on the EIT application in equine practice have focused on the assessment of ventilation in healthy standing horses [65,93]. Regional distribution of ventilation, the left-to-right lung region impedance ratios, and ventral-to-dorsal lung region impedance ratios were calculated. In healthy physiologically breathing horses, the right lung received a larger fraction of the tidal volume than the left, and the ventral-dependent lung region was more ventilated than the dorsal nondependent one [72]. Schramel et al. described the shift of regional ventilation towards dorsal nondependent regions in progressing pregnancy ponies that reversed seven days after foaling [95].

Application of EIT in anesthetized horses has been well described by Mosing et al. [71]. The EIT-based evaluation of the breathing pattern, distribution of ventilation, and gas exchange during anesthesia revealed the phenomenon of inspiratory breath-holding and the redistribution of gas from ventral to dorsal regions of the lung after recovery from general anesthesia [97]. The phenomenon of auto-recruitment by breath-holding has not been described previously and thus shed new light on horse anesthetized lung function. Mosing et al. also compared the lung function in spontaneously breathing and controlled mechanical ventilation anesthetized horses. In the spontaneously breathing horses, ventilation was essentially centered within dorsal regions of the lungs, while during controlled mechanical ventilation it shifted towards ventral regions [71]. Auer et al. repeated a similar protocol on the lateral recumbent ponies. The ventral shift of the ventilation region was explained by the loss of dorsal movement of the diaphragm when switching from spontaneous ventilation to controlled mechanical ventilation [98].

Moens et al., Wettstein et al., and Mosing et al. made an effort for a better understanding of horse ventilation by applying EIT measurements for continuous monitoring of the dynamic changes in the distribution of ventilation [71,93,99]. The EIT has been proposed as a monitoring tool in alveolar recruitment maneuvers in horses [93,97]. In alveolar recruitment maneuvers, lung opening is based on the implementation of sufficient peak inspiratory pressure and the immediate application of partial end-expiratory pressure [99]. These high airway pressures inevitably induce cardiovascular and pulmonary side effects such as decreases in cardiac output and blood pressure, as well as overdistension of lung parenchyma which results in augmented dead space fractions [93]. Therefore, positive end-expiratory pressure should be adjusted to the lowest pressure that prevents alveolar decruitment [93]. It is worth noting that the EIT-measured effects of the peak inspiratory pressure and partial end-expiratory pressure on the distribution of ventilation were in line with spirometry results in horses [73]. Moreover, the application of EIT in dorsally recumbent anesthetized horses allows for establishing the level of continuous positive airway pressure at which the number of silent spaces in the dependent parts of the lungs decreases [100]. However, to implement this ventilation strategy in clinical practice, the widespread awareness of the possible use of EIT or other appropriate monitoring tools in equine medicine needs to be greatly increased [101,102]. 

Among the direct clinical applications of the EIT in equine practice, recent research described the global and regional peak respiratory flows in the horses that underwent histamine challenges and drug-induced bronchodilatation [73,90], as well as those suffering from equine asthma [96]. The main component of equine asthma, a chronic disease that greatly affects the horse’s physical capacity, is airway inflammation that causes bronchoconstriction with recurring obstruction of air passages, excessive mucus production, and bronchial and pulmonary hyperresponsiveness [103]. It is worth noting that the EIT measurements proved to be effective in the evaluation of histamine-provoked bronchoconstriction in horses [73,90]. Horses were nebulized using histamine saline, and the total impedance change during inspiration and expiration, peak global inspiratory, and peak expiratory global flow were evaluated by calculating the first derivative of the EIT volume signal. In both studies, inspiratory and expiratory global EIT flow variables incrementally increased with bronchoconstriction. A reversal of airflow changes induced by the administration of albuterol after histamine challenge was also EIT detectable [73]. In subsequent studies, EIT was implemented to compare effort-dependent ventilation between horses with asthma and the healthy group. The global expiratory flow was significantly higher in horses affected with mild and severe asthma after 15 min of exercise of the lunge. In horses with airway obstruction, the breathing strategy changes, and the biphasic airflow pattern disappears [90]. In healthy horses, the normal breathing strategy is reflected by a biphasic inspiratory and expiratory airflow pattern [82]. In asthmatic horses, the increase in global flow is more pronounced during expiration, with an increase of 94% compared to inspiration during which an increase of 83% was observed. The EIT measurements proved that asthma affects expiration more than inspiration [65]. 

### 3.3. Impulse Oscillation System (IOS) Evaluation of Lower Respiratory Tract

The impulse oscillometry system (IOS) is a non-invasive effort-independent functional modality that allows for measuring both airway resistance (R) and airway reactance (X) [95]. The IOS gains a huge interest in pediatrics, wherein younger children’s ability to follow instructions is not required [104]. For a similar reason, the IOS successfully has been introduced into the veterinary field. In the equine application, the harmonic sound waves generated by a loudspeaker flow through the horses’ respiratory tract [66]. The harmonic sound waves may flow through the tube attached to the face mask during IOS examination on a standing, non-sedated horse or to the endotracheal tube during anesthesia. Therefore, the IOS may be applied in both clinical applications [75]. A loudspeaker generates the single or multiple frequency harmonic sound waves, which usually range from between 1 and 5 Hz to between 10 and 25 Hz. The impulses generated by the loudspeaker travel superimposed during normal tidal breathing through the large and small airways. Higher frequency harmonic sound waves penetrate out to the lung periphery, thus reflecting the large airways, whereas lower frequency waves travel deeper into the lung reflecting the lower airways. Finally, the inspiratory and expiratory flow and pressure are measured by the pressure and flow transducers [104]. The pressure and flow signals are separated from the breathing pattern by signal filtering. The signal coming back from the airways carries the data representing respiratory impedance which is the sum of all the resistance and reactance opposing the IOS-produced oscillations. The air resistance (R) is a force proportional to energy required to propagate the pressure wave through the airways, whereas the air reactance (X) is another force proportional to the amount of recoil generated against that pressure wave [104].

#### 3.3.1. Advantages

The IOS provides reliable, repeatable, and informative pulmonary functional data of the lower respiratory tract [75], completely different than the data obtained with other diagnostic techniques described. Therefore, it seems to be a very good complement, not a replacement for the basic and advanced diagnostic techniques of the equine respiratory tract. The IOS is non-invasive, fast, and easy to calibrate, and does not require the use of either ionizing radiation or electrical currents. Similar to spirometry and EIT, the equipment and facilities are affordable, portable, easy to implement, and well-tolerated by standing, non-sedated horses, thus the application of the IOS in the field conditions is promising [77]. Contrary to classic spirometry, the IOS requires only passive horse cooperation which is a great advantage in veterinary practice [77] and is the second indicator of applicability for field measurements in equine medicine. Cooperation with a qualified specialist is required during the first measurements conducted with the IOS, and online support of acquired data interpretation is also available [66].

#### 3.3.2. Disadvantages

Although the IOS has many useful promising clinical applications, the examination protocol is subject to some limitations. Despite the IOS protocol being effort-independent, the horse still needs to be cooperative, especially regarding wearing a mask that sometimes might be challenging and time-consuming [77]. Moreover, similar to spirometry, the horse masks need to be adjusted for each horse to avoid air leaks that may interfere with results. Thus, buying a wide spectrum of facemasks is needed to fit them properly to foals, small, and full-sized horses [66]. Maybe for this reason, despite the many advantages, the IOS is currently restricted to research institutions and referrals [66]. Moreover, similar to both spirometry and EIT, the IOS in equine medicine is still at an early stage of clinical development, and research remains ongoing regarding the protocols, interpretation, and clinical application in horses, although more reports are still needed [66]. 

#### 3.3.3. Clinical Applications

Van Erc et al. made a cornerstone in equine IOS application, providing the normal reference values for adult horses and standardizing the examination protocol concerning the effect of unfitted masks or position of the head. The horse’s gender, age, and general physical morphology determined by individual biometrics did not significantly affect the results of IOS measurements [75]. In other studies, van Erck et al. reported that bronchoconstriction resulted in an increase in R at 5 Hz and a decrease in X at 5, 10, 15, 20 Hz frequencies. Young et al. confirmed those results, however, at frequencies between 1 and 3 Hz [74]. Klein et al. investigated the effect of xylazine sedation on the IOS parameters and revealed significant alterations mainly during inspiration [76]. Richard et al. revealed the significant increase in R and decrease in X at lower frequencies 1–10 Hz in a horse group with mild equine asthma [105]. These promising results direct further research towards the assessment of the suitability of the IOS in the diagnosis of equine asthma at an early stage. Stucci et al. initiated the measurement of the Delta X (ΔX) in a horse’s respiratory tract, and defined ΔX as the difference between the inspiratory and expiratory reactance at each frequency [77]. In human medicine, ΔX is used in the detection of tidal expiratory flow limitation in chronic obstructive pulmonary disease and human asthma [106]. Based on the Stucci et al. results, ΔX could be used in monitoring the exacerbation or remission of the clinical signs of severe equine asthma and healthy controls, indicating the important clinical application of the IOS in the diagnostic and treatment of diseases of the lower equine respiratory tract [77].

## 4. Future Development and Practical Applications

In human medicine, specialized centers for respiratory diseases are present and the future may lead to development of similar facilities for horses, as respiratory problems highly impact the equids population. In many cases, the complexity of respiratory disorders makes the full diagnosis under field conditions inapplicable. Thus, specialized centers fully equipped to provide comprehensive diagnoses are needed. Except for the endoscopes and US machines, the remainder of the specialized equipment is not portable. Ambulatory X-ray systems are not suitable for thoracic radiography in adult horses, this area can only be achieved using high powered X-rays which are mainly stationary. Lung function tests are a very promising tool, especially regarding the worldwide occurrence of EA. Early asthmatic horses may not show recognizable signs, but their airway hyperactivity can be detected using a pulmonary function test. PFTs may be especially helpful in early diagnosis of high-performance horses where the smallest amount of respiratory disease may affect the future outcome. PFTs may be also helpful in the evaluation of response to treatment protocol and are more sensitive than BALF retake. Hopefully high demand from the owners and trainers will result in the wide implementation of those techniques in equine respiratory diseases diagnosis, as it would support effective management of affected horses, improve the outcomes, and improve the horses’ welfare.

## 5. Conclusions

Huge improvements have been observed in the last decades and continuous technical progress is changing the capability of the diagnostic techniques, allowing for more accurate anatomical and functional studies of the equine respiratory tract. Within the advanced techniques, the sensitive diagnostic modalities like CT or MR have provided the detailed anatomical features of upper respiratory tract diseases, allowing for the exact visualization of changes and providing vital information for surgical staff in the case of a planned procedure. The further implementation of pulmonary function tests is promising as a non-invasive tool to facilitate an earlier diagnosis of equine lower respiratory tract diseases, especially in the case of equine asthma. However, more studies in the presented field are needed to create protocols that may be widely implemented by equine practitioners.

## Figures and Tables

**Table 1:** The selected diseases of the equine head and neck area and their main findings diagnosed based on computed tomography imaging.

Disease	Area ^1^	Main Findings	Authors
Sinusitis	Paranasal sinuses	Thickening of the respiratory epithelium, teeth involvement. The inhomogeneous appearance of the thickened bone, sclerosis of the facial crest, deformed shape of the maxilla, irregularly defined periostitis, bone loss or perforation, soft tissue swelling of the face.	Henninger et al. (2003) [32] Tucker et al. (2001) [19]
Laryngeal dysplasia	Larynx	Thyroid cartilage abnormalities: lack of a cricothyroid articulation, a dorsal extension of the thyroid cartilage, absence of the caudal cornu of the thyroid cartilage, absence of the articular process of the cricoid cartilage, and hypoplasia or absence of the cricopharyngeus muscle.	Garrett et al. (2010) [43]
Cysts and cyst-like lesions	Larynx and cranial cervical trachea	Thickening, heterogenous signal intensity of thyroid cartilage laminae, the ventral and lateral aspects of the cricoid cartilage, and the ventral aspect of the first tracheal ring. The thyroid, arytenoid, and cricoid cartilages and the first tracheal ring, presence of focal areas of hyperintense signal consistent with fluid. Thickening of the nasal mucosa. Narrowed nasal meati. Homogeneous hyperintense signal, consistent with fluid of interior of the nasal septum.	Garrett et al. (2010) [43]
	Paranasal sinuses	Focal mineralization of the soft tissue mass. Fluid lines in one or more paranasal sinuses, dental apex flattening. Bulging and thinning of maxillary bone, partial destruction of the osseous orbit, infraorbital canal changes. Displacement and distortion of the osseous infraorbital or lacrimal canal.	Fenner et al. (2019) [33]
	Paranasal sinuses	Homogenous soft tissue/fluid filling the entire maxillary sinus. Expansion of right maxillary sinus with the erosion of the first molar.	Tucker et al. (2001) [19]
	Paranasal sinuses	Large, clearly demarcated mass within the left caudal maxillary and left conchofrontal sinuses. Lysis of the sphenoid and palatine bones of the medial left orbit and left infraorbital canal. Extension into the left retrobulbar space, with rostral and lateral displacement of the left globe.	Annear et al. (2008) [34]
Tumors	Paranasal sinuses	Squamosus cell carcinoma: irregularly surfaced heterogeneous soft tissue mass filling the maxillary sinus and ventral conchal sinus.	Kowalczyk et al. (2011) [39]
	Paranasal sinuses, nasal cavity, tongue, mandible	Squamosus cell carcinoma: soft tissue attenuation filling maxillary sinus, dorsal conchal sinus, ventral conchal sinus, while the conchofrontal and sphenopalatine sinus showed different amount of filling. Nodular masses involved a third of the ipsilateral rostral maxillary sinus and less than a third of the conchofrontal sinus. Involvement of stylohyoid bone. Small nodular soft tissue lesions along the nasal septum.	Strohmayer et al. (2020) [28]
	Neck	Dystrophic mineralized mass at the right side of the vertebral bodies of C3 and C4, associated with bone resorption that caused the thinning of the right transverse process and a widening of the angle between the transverse process and the arch of C3.	De Zani et al. (2011) [30]
	Nasal cavity, Paranasal sinuses	Hemangiosarcoma, nasal adenocarcinoma, myxoma, myxosarcoma, chondroblastic osteosarcoma, anaplastic sarcoma characterized by a homogeneous, poorly defined mass that was iso- or mildly hypoattenuating compared to masseter muscle.	Cissel et al. (2012) [31]
	Paranasal sinuses	Osseous fibroma: well-marginated mass in right nasal passage with destruction of caudal aspect of nasal septum and extension of the mass into the choanae. Rostrocaudal extent of the soft tissue density with loss of bone density in the vicinity of the cribriform plate	Cilliers et al. (2008) [29]
Temporohyoid osteoarthropathy	Temporohyoid articulation	Osseous proliferation of the stylohyoid bone and temoporohyoid articulation, thickening of ceratohyoid bone. Lytic osseous changes of the petrous temporal and stylohyoid bones.	Hilton et al. (2009) [36] Divers et al. (2006) [37] Bras et al. (2014) [38]

^1^ Area of the respiratory tract; CT—computed tomography.

**Table 2:** The selected diseases of the equine head and neck area and their main findings diagnosed based on magnetic resonance imaging.

Disease	Area ^1^	Main Findings	Authors
Cyst	Paranasal sinus	Homogeneous On T1W sequences, the cyst was hypointense compared to temporal muscles, no contrast enhancement within the cystic fluid. rim enhancement On T2W sequences, the contents were hyperintense to surrounding muscle, and a wall could be observed consistently surrounding the lesion. This rim was observed consistently around the lesion and could be differentiated from the adjacent mucosa.	Tessier et al. (2013) [54]
Abscess	Ventral Conchal sinus	Well-defined capsule with heterogeneous signal intensity in T2W images, deviation of the dorsal conchal sinus wall, and infraorbital canal.	Manso Diaz et al. (2015) [42]
Tumors	Nasal septum	Chondrosarcoma: heterogeneous intensities on all sequences and no defined borders of the lesion.	Tessier et al. (2013) [54]
	Middle nasal meatus	Osteoma: irregularly shaped mass that was hypointense on both T1W and T2W images, containing small foci isointense to muscle on T2W images, maxillary bone atrophy.	Manso Diaz et al. (2015) [42]
	Nasal cavity	Lymphoma, squamous cell carcinoma: expansile, heterogeneously, and moderate contrast-enhancing mass with complete occlusion of the nasal cavity.	
Laryngeal dysplasia	Larynx	Lack of a cricothyroid articulation, dorsal extension of the thyroid cartilage, absence of the caudal cornu of the thyroid cartilage, absence of the articular process of the cricoid cartilage and hypoplasia, or absence of the cricopharyngeus muscle.	Garrett et al. (2009) [55]

^1^ Area of the respiratory tract; MR—magnetic resonance, T1W—T1 weighted image, T2W—T2 weighted image.

**Table 3:** The selected diseases of the equine lung area and their main findings diagnosed based on the consecutive pulmonary function tests.

Function	Technique	Area ^1^	Main Findings	Authors
Monitor ventilator volumes and respiratory mechanics, control the depth of the anesthesia	Spirometry with pilot-based flow meter	Lungs	Measurement of tidal volume and minute volume, dynamic compliance (Cdyn) of the respiratory system. Visual presentation of pressure-volume (PV) and flow-volume (FV) loop of each breath, representing the compliance (PV) and resistance (FV) of the respiratory system.	Moens et al. (2010) [67]
Correlation of spirometry results and percentage of neutrophils (N%) in tracheal aspirates	Spirometry	Lungs	Wide variation in N% in tracheal aspirates of clinically normal horses with poor racing performance, spirometry results significantly correlated with measurements of N% in tracheal aspirates.	Evans et al. (2011) [68]
Comparison of tidal breathing flow-volume loop (TBFVL) of healthy horses and horses suffering from mild and to severe asthma	Spirometry	Lungs	Disease-related differences in TBFVL indices are affected by the type of work undertaken by a horse.	Herholz et al. (2003) [69]
Measurement respiratory rate, tidal volume, peak inspiratory and expiratory flows, time to peak flow in healthy horses	Spirometry	Lungs	Measurements were repeatable and reproducible, however variable breathing patterns within the same day and on a breath-to-breath basis were present.	Burnheim et al. (2016) [64]
Effect of sedation and salbutamol administration on tidal breathing	Spirometry	Lungs	After sedation, minute ventilation was reduced in association with reduced respiratory rate and decreased expiratory and inspiratory flows. Relative expiratory time was reduced after xylazine, and peak expiratory flow occurred later in the respiratory cycle. Salbutamol administration has a significant effect on most parameters except the increase in peak inspiratory flow during tidal breathing.	Raidal et al. (2017) [70]
Monitoring of ventilation during anesthesia	EIT	Lungs	Inspiratory breath-holding and the redistribution of gas from ventral to dorsal regions of the lung after recovery from general anesthesia.	Mosing et al. (2016) [71]
Monitoring of recruitment maneuvers (RM) during anesthesia	EIT	Lungs	During recruitment maneuvers, ventilation in independent ventral region.	Ambrisco et al. (2015) [72]
Detection of bronchoconstriction and bronchodilatation	EIT	Lungs	EIT-derived flow indices for ventilation significantly changed after histamine administration and returned to control values with subsequent albuterol administration.	Secombe et al. (2021) [73]
Effect of sedation and salbutamol administration on tidal breathing				
Diagnosis and monitoring of equine asthma	EIT	Lungs	Healthy horses have lower peak expiratory and inspiratory flow compare to horses with mild or severe asthma after exercise.	Herteman et al. (2021) [65]
Diagnosis of bronchoconstriction	IOS	Lungs	IOS parameters in the low-frequency range were sensitive indicators of early methacholine-induced bronchoconstriction.	Van Erck et al. (2003) [74]
Standardization of IOS measurements	IOS	Lungs	IOS measurements were reliable and repeatable. Age, sex, and bodyweight did not influence IOS measurements. Measurements from 5 to 15 Hz were found to be most relevant.	Van Erck et al. (2004) [75]
Effects of sedation on lung airflow	IOS	Lungs	Inspiratory parameters were found to be significantly dependent on the time course of sedation, whereas expiratory parameters were not influenced.	Klein et al. (2006) [76]
Diagnosis and staging of equine asthma	IOS	Lungs	Significant changes were present between horses in exacerbation of EA and control horses within inspiratory and expiratory parameters. The delta reactance (ΔX) shows the presence of tidal expiratory flow limitation (EFLt) and dynamic airway compression in SEA horses in exacerbation of the clinical signs.	Stucchi et al. (2022) [77]

^1^ Area of the respiratory tract; EIT—electrical impedance tomography; IOS—impulse oscillometry system.

## Data Availability

Not applicable.
